# Genetic architecture of sex determination in fish: applications to sex ratio control in aquaculture

**DOI:** 10.3389/fgene.2014.00340

**Published:** 2014-09-29

**Authors:** Paulino Martínez, Ana M. Viñas, Laura Sánchez, Noelia Díaz, Laia Ribas, Francesc Piferrer

**Affiliations:** ^1^Departamento de Genética, Facultad de Veterinaria, Universidad de Santiago de CompostelaLugo, Spain; ^2^Departamento de Genética, Facultad de Biología, Universidad de Santiago de CompostelaSantiago de Compostela, Spain; ^3^Institut de Ciències del Mar, Consejo Superior de Investigaciones CientíficasBarcelona, Spain

**Keywords:** sex determination, fish, genetic architecture, sex ratio, aquaculture

## Abstract

Controlling the sex ratio is essential in finfish farming. A balanced sex ratio is usually good for broodstock management, since it enables to develop appropriate breeding schemes. However, in some species the production of monosex populations is desirable because the existence of sexual dimorphism, primarily in growth or first time of sexual maturation, but also in color or shape, can render one sex more valuable. The knowledge of the genetic architecture of sex determination (SD) is convenient for controlling sex ratio and for the implementation of breeding programs. Unlike mammals and birds, which show highly conserved master genes that control a conserved genetic network responsible for gonad differentiation (GD), a huge diversity of SD mechanisms has been reported in fish. Despite theory predictions, more than one gene is in many cases involved in fish SD and genetic differences have been observed in the GD network. Environmental factors also play a relevant role and epigenetic mechanisms are becoming increasingly recognized for the establishment and maintenance of the GD pathways. Although major genetic factors are frequently involved in fish SD, these observations strongly suggest that SD in this group resembles a complex trait. Accordingly, the application of quantitative genetics combined with genomic tools is desirable to address its study and in fact, when applied, it has frequently demonstrated a multigene trait interacting with environmental factors in model and cultured fish species. This scenario has notable implications for aquaculture and, depending upon the species, from chromosome manipulation or environmental control techniques up to classical selection or marker assisted selection programs, are being applied. In this review, we selected four relevant species or fish groups to illustrate this diversity and hence the technologies that can be used by the industry for the control of sex ratio: turbot and European sea bass, two reference species of the European aquaculture, and salmonids and tilapia, representing the fish for which there are well established breeding programs.

## INTRODUCTION

Fish represent the most diverse group of vertebrates including more than 28,000 species (Nelson, 2006). This diversity is a reflection of their high capacity for adaptation to a broad spectrum of environmental conditions. As a result, fish show amazing morphological, physiological, and behavioral adaptations to live in the highly diverse aquatic environment. Fish also show all types of reproductive strategies, including gonochorism, proterandrous, protogynous, and simultaneous hermaphroditism, and unisexuality (Devlin and Nagahama, 2002). These reproductive strategies emerged independently in different lineages during evolution demonstrating a polyphyletic origin (Avise and Mank, 2009).

Domestication of fish for production is an ancient practice and again shows the high adaptation capacity of this group, especially considering that more than 354 fish species are cultivated all over the world (Food and Agriculture Organization [FAO], 2014). Production of domestic fish largely relies on reproduction, and a vast amount of information has been gathered for its control. Reproduction techniques include production of monosex populations because the existence of sexual growth dimorphism, either in favor of males or females depending on the species, and also because sometimes the most valuable trait is associated with one sex (e.g., color, shape, secondary sexual ornaments).

Here, we review the available data on the genetic architecture of sex determination (SD) in fish and how sex ratio is controlled in aquaculture production. We contrast this information with models emerging from the classical studies in *Drosophila*, mammals, or birds with highly conserved mechanisms associated to marked sex chromosome heteromorphisms. We show the huge intra and interspecific diversity of SD systems in fish associated to a high evolutionary turnover. So, its genetic architecture, although commonly supported by major genes, is also influenced by minor genes, and environmental factors approaching it to a complex trait. We illustrate this diversity and its influence on the strategies aimed at the production of the most desired sex in the last section of the paper by analyzing the genetic basis of SD in two important species of the European aquaculture, turbot (*Scophthalmus maximus*), and European sea bass (*Dicentrarchus labrax*). We also consider two of the main fish groups with established breeding programs, salmonids, and tilapias, all of them with remarkable differences in sex determination.

## GENETIC ARCHITECTURE OF SEX DETERMINATION IN FISH

The genetic architecture of a complex trait refers to the genes involved in that trait, their influence and interactions to establish the phenotype. It takes also into account the influence of environmental factors and their interactions with the genotype on the final phenotype (McKay, 2001). Although getting all this information is a very ambitious enterprise, the more we approximate to this goal the better we shall understand key questions related to the genetic variation for its exploitation in genetic breeding programs.

### MAIN FEATURES OF GONAD DEVELOPMENT

**FIGURE 1:**
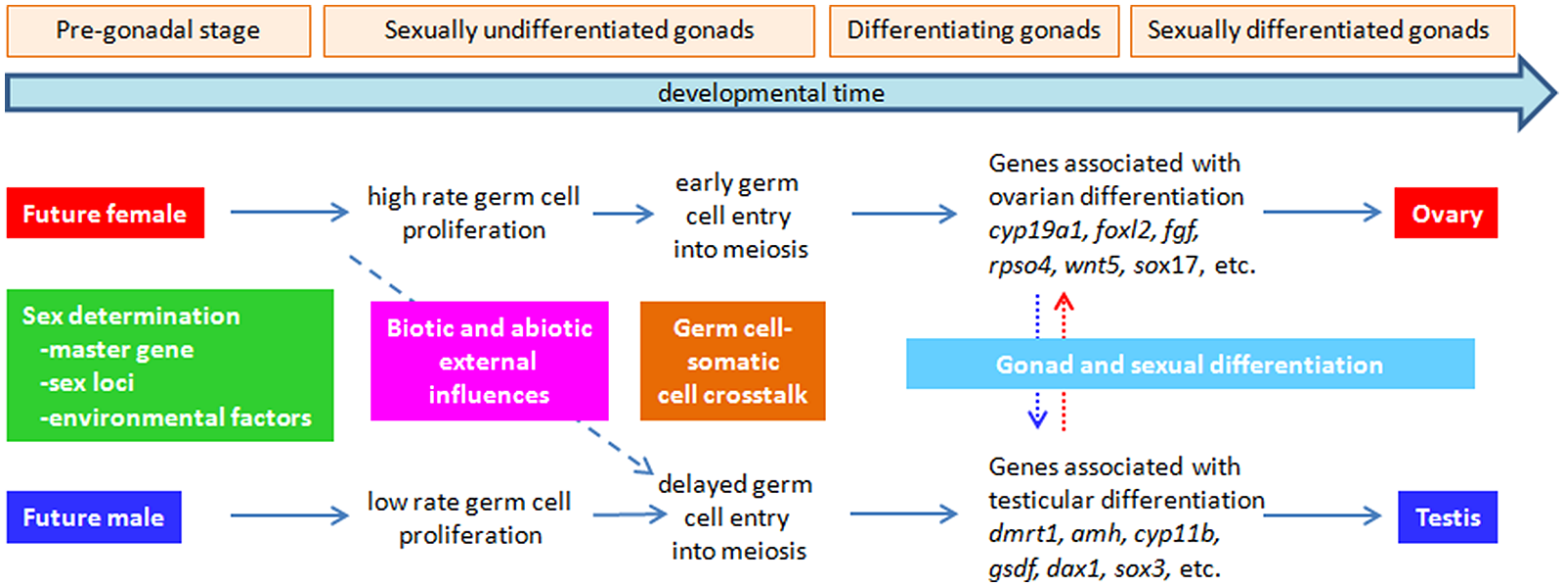
**Major events leading to ovarian vs. testicular differentiation in fish.** The first event, sex determination – the establishment of gender – can be triggered by the action of a major sex determining master gene, several sex-associated loci, an environmental factor (e.g., temperature) in an ecologically relevant context (i.e., occurring normally in the habitat of the species) or a combination of them, typically when the gonads are still sexually undifferentiated or even before they are formed at the most rudimentary stage (pre-gonadal stage). Successive events are connected by horizontal arrows and include differences in the proliferative rate of germ cells (females > males), which can be one of the first effects of the sex determining factor, whether genetic or environmental. During this period, the germ cell-somatic cell crosstalk is very important, but still largely uncharacterized. Also, during these early events, biotic and abiotic factors (e.g., stress, abnormally high temperature, etc.) can change the course of subsequent sex differentiation, usually in the female → male direction (diagonal dashed arrow). Finally, also at the beginning of gonad differentiation – the transformation of an undifferentiated gonad into a testis or ovary – the accidental (i.e., contamination) or deliberate (e.g., sex control treatment) incorporation of sex steroids, androgens or estrogens can result in female → male (vertical blue dotted arrow) or male → female (vertical red dotted arrow) sex-reversal, i.e., in that genotypic females and males develop into phenotypic males and females, respectively.

Gonad development includes all developmental processes aimed to transform an undifferentiated primordium into a mature gonad, either ovary of testis. It is the result of two concatenated development processes controlled by a hierarchical genetic network: SD and gonad differentiation (GD; **Figure 1**). The sex of gonads is essentially determined by processes, either genetic or environmental, operating at the beginning of development, where a binary decision is taken related to the fate of the undifferentiated primordium (Kobayashi and Nagahama, 2009; Siegfried, 2010; Uller and Helanterä, 2011). Once the future of the gonad has been established, morphogenetic GD processes work until maturation is completed.

As a consequence of the hierarchical nature of gonad development, a single gene or environmental cue operating at the beginning of development can drive the gonad pathway towards one direction or another. Thus, in contrast to other characters influenced by many genes operating in different routes with important additive effects, here gene interactions likely represent an important genetic component. Particularly, epistatic effects may be relevant because a single gene acting upstream or even downstream of a preexisting SD gene (SDg) may take the control of gonad fate, thus, masking extant genetic variation at other involved loci. Epistatic interactions have been reported between major SD loci in different fish groups (Cnaani et al., 2008; Ser et al., 2010; Parnell and Streelman, 2013), and also epistatic allelic variants have been reported segregating in populations of species with a well known SD genetic system like medaka (*Oryzias latipes*; Shinomiya et al., 2010). In fact, notable interactions occur between gene products at the initial stages of gonad development such as the suppression of *wnt4* and β*-catenin,* key genes for ovarian development, by *sox9* and *fgf9* (Nef and Vassalli, 2009); the modulation of gonadal aromatase (cypb19a), responsible of the balance between androgens and estrogens, by the action of other genes or environmental factors such as temperature (Navarro-Martín et al., 2011); or the interaction between the anti-müllerian hormone (amh1) and its receptor (amhr2), which triggers an essential signaling pathway for testis development (Kamiya et al., 2012).

Gonad development of fish is unusual in the sense that the sexually undifferentiated period can last from weeks until years (Saito and Tanaka, 2009; Berbejillo et al., 2012) opening a large developmental window in which the sexual fate can be influenced by abiotic or biotic environmental factors (Penman and Piferrer, 2008; Baroiller et al., 2009). In such a long period, it is tempting to speculate that the brain may be involved through the hypothalamic–pituitary–gonadal axis (Baroiller et al., 2009). However, despite the fact that the brain certainly integrates environmental stimuli and, in particular, social interactions, which have been shown to be implicated in the process of sex-change in hermaphrodites, currently there is no convincing proof that the brain plays any role in the SD process in gonochoristic fishes.

Homoeothermic vertebrates show a conserved morphogenetic development supported by a strongly canalized genetic sex determining system (Charlesworth et al., 2005). However, fish show inter-specific differences in the morphogenetic events occurring along gonad development. Variation exists both regarding the general pattern of differentiation, the interaction between somatic and germ cells, and in the time of occurrence and relative weight of the different steps. The amount of primordial germ cells have been reported to be the first development difference between males and females in species such as medaka (*O. latipes*; Kondo et al., 2009) and stickleback (*Gasterosteus aculeatus*; Lewis et al., 2008), and this has been related to the possible influence of growth-related factors on SD (Schlueter et al., 2007; Baroiller et al., 2009). Also, gonad development has been classified as undifferentiated or differentiated type, respectively, depending on the existence of an initial transitory female stage that can subsequently revert to testis like in zebrafish (*Danio rerio*; Orban et al., 2009), or the lack of that stage like in medaka or tilapia (*Oreochromis niloticus*; Saito and Tanaka, 2009). Interaction between somatic and germ cells is also recognized as an important feature for gonad development. In fact, several key genes related to the initial steps of differentiation like *dmrt1*, *amh*, or *sox9* in males (Lee et al., 2009; Wu et al., 2010) and *cyp19a1a* or *foxl2* in females (Nakamura et al., 2009) are expressed in Sertoli or granulosa/theca cells, respectively, and thus, communication between somatic and germ cells is essential for GD. In this communication there are species-specific differences and, for example, the ablation of the female germ cells determines the reversal of development towards a testis in zebrafish (*D. rerio*) and tilapia (Siegfried and Nusslein-Volhard, 2008; Fujimoto et al., 2010), while in goldfish (*Carassius auratus*) the female pathway is maintained (Goto et al., 2012).

### SEX DETERMINATION: ORIGIN AND EVOLUTION

A consensus existed until recently on the high conservation of the gene network controlling gonad development among vertebrates, differences being mainly related to changes in the switching mechanism. This hierarchical development controlled process would facilitate the control of sex ratio by a single-gene mechanism, but at the same time it would open the opportunity for changing the SD factor in response to new evolutionary scenarios (Marín and Baker, 1998).

**FIGURE 2:**
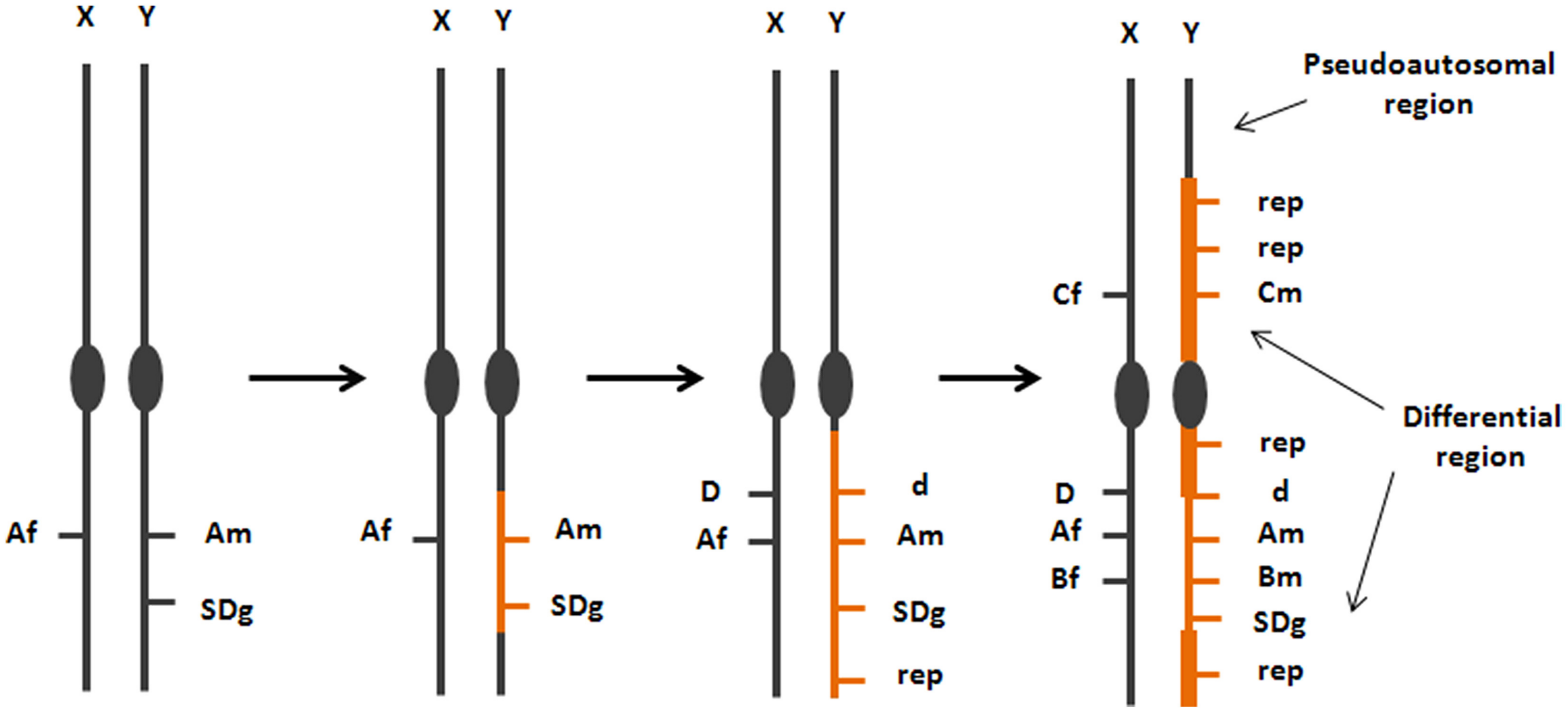
**Model on the origin and evolution of the SD region-bearing chromosome from studies in mammals, birds and *Drosophila*.** This model, although has been demonstrated in some fish, shows a large variation on its progression, which is reflected on the degree of differentiation between the chromosomes of the sexual pair. The origin of a new sexual pair is related to the origin of genes (A,B) with antagonistic alleles favorable to females (Af, Bf) or to males (Am, Bm) associated with a new SDg. Subsequent steps involve accumulation of repetitive elements (rep) and the degeneration of the Y chromosome because of its permanent heterozygotic state at the differential region (d: recessive non functional variant of a sex-linked gene).

Theories on the evolution and genetic architecture of SD in animals have been largely influenced by studies on *Drosophila*, mammals, and birds, all of them showing convergent patterns, with a heteromorphic sex chromosome pair and, as a consequence, a particular sex-linked inheritance model (Bachtrog et al., 2014). A generalized theory on the origin and evolution of SD systems emerged from these data, which assumed a sexual conflict between antagonistic alleles at specific loci favorable to one sex but detrimental to the other (**Figure 2**; Charlesworth et al., 2005). To maintain the beneficial association between the antagonistic allele and the SD locus, recombination would be restricted, giving rise to the permanent heterozygous state of that portion of the sexual pair (Bergero and Charlesworth, 2009). That circumstance would promote the accumulation of repetitive elements and deleterious variants in the SDg-bearing chromosome, contributing to its progressive degeneration and the typical heteromorphic shape of the sexual pair (Charlesworth et al., 2005). Mathematical models based on this theory suggested that only one gene should underlie the SD system, and if more than one gene were segregating, this should represent an unstable equilibrium towards a new SD mechanism (Rice, 1986).

Initial data in ectothermic vertebrates, particularly fish, demonstrated a sharply different picture (Devlin and Nagahama, 2002). More than one sex-associated gene has been reported in many fish species (Cnaani et al., 2008; Ser et al., 2010). In addition, the conservation of the GD cascade has been to some degree questioned, and notable genetic differences have been observed not only at the top, but also downstream of the GD network (Böhne et al., 2013; Herpin et al., 2013). For example, the well-known female-associated aromatase genes (*cyp19a1a* and* cyp19a1b*) have shown a new role in testis differentiation in African cichlids (Böhne et al., 2013). Although antagonistic alleles demonstrated to be associated with the SD region in fish (Roberts et al., 2009; Parnell and Streelman, 2013), only 7% species showed heteromorphic sex chromosomes (Penman and Piferrer, 2008; Oliveira et al., 2009). This has been related to the huge evolutionary turnover of SD mechanism which limits chromosome differentiation and, as a consequence, different SD systems have been reported between closely related species and even between populations of the same species. However, degeneration and differentiation of sex chromosomes can evolve very quickly (Charlesworth et al., 2005) and a broad heteromorphism degree has been observed among species of the same genus in Neotropical fish of the genera *Eigenmannia* (Henning et al., 2011), *Characidium* (Vicari et al., 2008), and *Leporinus* (Parise-Maltempi et al., 2013). Thus, sex heteromorphism can involve the whole chromosome and be detectable with the usual cytogenetic techniques, as reported in Neotropical fish (Henning et al., 2011; Parise-Maltempi et al., 2013); be cryptic at cytogenetic level but involving an important degeneration of the SDg-bearing chromosome, as in stickleback (Ross and Peichel, 2008; Shikano et al., 2011); embrace no more than a few kilobases as in medaka (*O. latipes*; Matsuda et al., 2002); or show a very tiny differentiation as the single SNP observed in the *amhr2* receptor, the only detectable difference between X and Y chromosomes in fugu (*Takifugu rubripes*; Kamiya et al., 2012).

To understand the origin of SD regions both external pressures (i.e., sexual selection) and the internal context (available genes and genomic structure) should be considered. Several genes with different functions have been recruited along evolution as SDg in fish, which shows the opportunistic nature of selection to face new evolutionary pressures. In this regard, the specific genome duplication occurred within teleosts may have provided a suitable raw material for new sex determinants (Mank and Avise, 2009). A remarkable case which exemplifies the many options available to change SD mechanisms is the association of sex to B chromosomes, usually considered as junk DNA, in species of the genus *Astyanax* and in two species of pufferfish (Vicente et al., 1996; Noleto et al., 2012). The high turnover of the SD region in fish has led to suggest that changes in the SD mechanism may be associated with speciation (Ser et al., 2010; Böhne et al., 2013; Parnell and Streelman, 2013).

### THE GENETIC BASIS OF SEX DETERMINATION IN FISH

High genetic variation has also been described between fish species regarding the gene responsible for SD, the number of genes involved in such decision and the relationships between them. Currently, five different master genes have been documented in fish: *dmY*, *gsdf*,* amhy*, *amhr2*, and *sdY*. *dmY* (DM-domain gene on the Y chromosome), the SDg of medaka and the first one described in fish (Matsuda et al., 2002), is a transcription factor expressed in the somatic cells surrounding germ cells before sex differentiation and in the testis thereafter and it is involved in germ cell proliferation and development of pre-Sertoli cells into Sertoli cells. It originated from a segmental duplication of a small autosomal region containing the precursor *dmrt1*, followed by an insertion of the duplicated region on the proto-Y chromosome (Matsuda et al., 2002). *gsdf*,* amhy*, and *amhr2* are members of the TGF-ß superfamily involved in cell signaling controlling cell proliferation (Heule et al., 2014). *gsdf* (gonadal soma derived growth factor) is a downstream gene of *dmY* in the SD cascade that has taken the role of master SDg in *Oryzias curvinotus* (Myosho et al., 2012). *amhy* (Y chromosome-specific anti-müllerian hormone) is expressed in the presumptive Sertoli cells of XY males of *Odonthestes hatcheri* during the onset and subsequent GD. This gene has been inserted upstream of *amh* in the cascade of male development, becoming a male SDg (Hattori et al., 2012). In *T. rubipres*, *amhr2* (anti-müllerian hormone receptor type 2) is expressed in somatic cells surrounding germ cells and it is thought to be the SDg in this species. This gene contains a specific SNP variant in the kinase domain of *amhr2* in the X chromosome which determines lower affinity for the amh hormone, thus fating the female pathway when homozygous (XX; Kamiya et al., 2012). Finally, *sdY* (sexually dimorphic on the Y chromosome) is linked to the SEX locus of salmonids and is necessary and sufficient to induce testicular differentiation. It has evolved through neofunctionalization from *irf9* by losing its role in *ifn* signaling pathway and acquiring a new role in SD (Yano et al., 2012).

From an evolutionary perspective different SDgs have been reported either in closely related species like *O. latipes (dmY)* and *Oryzias luzonensis* (*gsdf1*; Myosho et al., 2012) or in divergent ones such as rainbow trout (*Oncorhynchus mykiss*; *sdY;* Yano et al., 2012), fugu (*amhr2*; Kamiya et al., 2012), or pejerrey (*O. hatcheri*; *amhY*; Hattori et al., 2012). Also, the same SDg has been identified in closely related species like* O*.* latipes* and *O. curvinotus* (*dmY*; Matsuda et al., 2002); *T. rubripes, Takifugu pardalis*, and *Takifugu poecilonotus* (*amhr2*; Kamiya et al., 2012); and in most salmonid species studied so far (*sdY*; Yano et al., 2013). Very recently, *dmrt1* has been suggested as a strong candidate in the half-smooth tongue sole (*Cynoglossus semilaevis*) based on its association with sex and its pseudogenization in the W chromosome (Chen et al., 2014). This would constitute the first SDg reported in a ZW system species and within flatfish, a group of species of great commercial value and with a particular metamorphosis to adapt to demersal life. However, no functional demonstration has been reported to date thus requiring further investigation.

Additional information exists from marker and QTL sex-associated studies which are usually unraveling variation on major genes controlling the fate of the undifferentiated primordium, and thus, basically related to SD. The relationships between the different genomic regions identified through this approach has been established through comparative mapping using model fish as a bridge, taking advantage of the conserved macrosynteny pattern observed in teleosts (Kai et al., 2011; Bouza et al., 2012). In most fish groups analyzed, these SD regions demonstrated to be non-homologous (Piferrer et al., 2012), so in the *Oryzias* (medaka) genus up to five different genes/genomic regions seem to be involved in sex determination, and only two species among the six analyzed show the same SDg (Tanaka et al., 2007); in the Gasterosteidae (stickleback) family four different genomic regions have been identified in the five species analyzed, and in the fifth, a fusion of two previously reported SD chromosomes gave rise to the sexual chromosome (Ross et al., 2009); in the tilapiine (tilapia) cichlid tribe two chromosomes have been identified [linkage group 1 (LG1) and LG3], two species associated with LG1, other two with LG3, and the remaining two showing both linkage groups, but other studies also demonstrated association with LG23 (Cnaani, 2013); in the Salmoniformes (salmonids) order most species showed different non-homologous sex-associated genomic regions (Phillips et al., 2001); and finally, in the Poeciliidae (guppy and platyfish) family, up to four different chromosomes are involved in SD (Tripathi et al., 2009). Very likely these non-homologous regions include different SDgs, although in salmonid species showing non-homologous SD genomic regions, the SDg appears to be the same (Yano et al., 2013).

The diversity of SDg in fish highlights the many options available at the undifferentiated stage of gonads to switch and drive gonad fate, although some genes have been recurrently used because of their prominent position in the development cascade (Graves and Peichel, 2010; Heule et al., 2014). Among them, *dmrt1* and related genes found in medaka and likely in half-smooth tongue sole have also been reported in different vertebrates, including birds and amphibians (Smith and Sinclair, 2004; Yoshimoto et al., 2008), which illustrate processes of convergent evolution related to the suitability of some genes acting at the beginning of development. Furthermore, a great plasticity has been shown, since it can be found in XY or ZW systems and acting on a presence/absence model (medaka and frog) or in a dose-dependent manner (birds and likely tongue sole; Koopman, 2009; Chen et al., 2014). Three other genes, *gsdf1*, *amh1*, and *amhr2*, have also been reported to be activated at the beginning of development in the male pathway, and thus their SD role fits to the top of the GD invoked by theory (Heule et al., 2014). Contrary to previous findings in vertebrates (*sry* and *dmrt-*derived genes), *gsdf1*, *amh1*, and *amhr2* are not transcription factors being involved in cell signaling controlling cell proliferation (Heule et al., 2014). Finally, the *sdY* gene, the major SD factor in rainbow trout, represents an unexpected SDg of unknown function, whose carboxi-terminal extreme is homologous to an interferon-related gene, thus exemplifying the vast source of genes available for leading the SD process (Yano et al., 2012).

In addition to species with a single major SDg, many fish have shown more than one gene of big effect involved in sex determination. Indeed, in the most investigated fish groups at least two major genes (or genomic regions) related to SD have been reported in the same species: within tilapiinid, two major male and female determinant genes on LG1 and LG3, respectively, (Cnaani et al., 2008); in the platyfish, a poecilid species, a multifactorial SD system with X,Y, and W sex chromosomes (Schultheis et al., 2009); in the sticklebacks, *Gasterosteus weathlandi* a multiple chromosome system due to the fusion of two major SD chromosomes presented in other species of Gasterosteidae (Ross et al., 2009); and in cichlids from lake Malawi, two main SD systems on LG5 and LG7, the first one representing a female epistatically dominant factor (Ser et al., 2010). Finally, a polygenic SD system has also been documented in other species like European sea bass (Vandeputte et al., 2007) and zebrafish (Liew et al., 2012)

The lowering cost of new generation sequencing (NGS) methodologies will allow obtaining much more information in the near future to get a more accurate picture of SD in fish. Restriction-site associated DNA (RAD) sequencing, a technique which combines the powerful of NGS with the simplification of genomes through restriction enzyme digestion (Baird et al., 2008; Davey et al., 2011), is enabling to perform dense genomic screening to study the genetic architecture of multigene traits (Hohenlohe et al., 2010). This methodology is being applied for the study of SD in zebrafish (Anderson et al., 2012) and to identify sex-associated genomic regions in Nile tilapia (*O. niloticus*; Palaiokostas et al., 2013a) and Atlantic halibut (*Hippoglossus hippoglossus*; Palaiokostas et al., 2013b) for its application in aquaculture production. In addition, the high capacity of RAD sequencing for SNP discovery and constructing genetic maps will aid to get dense maps at candidate regions to narrow them and facilitate the identification SDgs (Taboada et al., 2014).

Gene expression studies linked to NGS methodologies will also be essential to understand the relationship between morphogenetic effects and the underlying genetic network. Microarrays have been used as a powerful tool for assessing gene expression profiles along GD (Gardner et al., 2012; Sreenivasan et al., 2014), but more recently, RNA-Seq is being applied due to its higher sensitivity, accuracy, and also because it provides additional information on genetic variants linked to expression differences (Sun et al., 2013; Tao et al., 2013). Finally, the evaluation of the pattern of methylation through bisulfite sequencing and other methodologies is providing quick genomic evaluation of the epigenomic maps along development (Cokus et al., 2008) constituting a valuable tool for understanding the regulation of SD and gonadogenesis (Piferrer, 2013).

### ENVIRONMENTAL FACTORS ON SEX DETERMINATION

In environmental sex determination (ESD), the first difference between the two sexes is established by differences in the value of an environmental factor. ESD has been well studied in reptiles like crocodiles and turtles (Valenzuela and Lance, 2004). In *Menidia menidia* (Conover and Kynard, 1981), the first fish species where temperature-dependent sex determination (TSD), a form of ESD, was first described, some populations have shown a genetic component underlying SD (Conover and Heins, 1987). Much of the literature in this field demonstrated the influence of environmental factors in laboratory conditions, extreme in some cases, but not necessarily reflecting the conditions that species experience in nature (Ospina-Álvarez and Piferrer, 2008). Nevertheless, the presence of TSD in fish demonstrates the plasticity of gonad development (Baroiller et al., 2009).

Social interactions represent important environmental cues for SD in hermaphroditic species (Godwin, 2009). In these cases, the brain has shown to be a major player translating social cues into a physiological signal (Kobayashi et al., 2010). Although this is not a mechanism driving the gonad fate at the beginning of development, it is a good example of the plasticity of gonad development in fish and also an evidence on the existence of bipotential primordium cells in the differentiated gonads of adult fish (Zhou and Gui, 2010).

Temperature is the environmental factor with highest influence in SD in fish (Ospina-Álvarez and Piferrer, 2008; Baroiller et al., 2009). Temperature influence on sex shifting can be exerted at several points of the differentiation cascade. High temperatures usually tend to produce more males and low temperatures have no effects or produce more females in some cases (Ospina-Álvarez and Piferrer, 2008). The ultimate mechanism (if there were a single one) connecting temperature and sex ratio is not known, and several have been proposed. The influence of temperature on SD has been related to a higher stress, giving rise to changes in circulating cortisol levels. In fact, the administration of cortisol in the diet has demonstrated a significant influence on sex ratio (Mankiewicz et al., 2013). However, it is far from clear the ultimate molecular mechanism connecting cortisol levels and masculinization. On one hand, Hayashi et al. (2010) proposed a direct up-regulation of the follicule stimulant hormone (FSH) receptor, which would be connected to germ cell proliferation. On the other, Fernandino et al. (2013) suggested an up-regulation of hsd11b2, a steroidogenic enzyme implicated both in the metabolism of cortisol into cortisone, and in the synthesis of biologically active androgens such as 11-ketotestosterone. Epigenetic regulation of aromatase expression mediated by temperature has also been proposed. Thus, Navarro-Martín et al. (2011) demonstrated that hypermethylation of aromatase promoter is correlated with high temperature during the thermosensitive period in the European sea bass, strongly suggesting that sex differentiation is under epigenetic control in this species.

### GENETIC VARIATION WITHIN SPECIES: SEX DETERMINATION AS A COMPLEX TRAIT

Most studies in SD in fish have been focused on identifying the master SDg expected according to previous SD models. However, new data are consistently showing that other minor genetic and environmental factors are also involved in SD, even in species with well studied master genes like *dmY* of medaka (Matsuda et al., 2002), and thus, more effort should be devoted to investigate this variation to get the closest picture as possible on SD in fish.

The information exposed so far shows that: (i) although environmental factors may influence sex ratio, usually the genetic component represent the main SD factor in most studied species; (ii) major genes, those which explain a high proportion of the trait phenotypic variance, are on the basis of SD in many species, likely an expected fact, because of the hierarchical nature of the sex development pathway; and (iii) the interaction of other genes involved in SD with the major factors and the environment. Although major genes are involved in SD of many fish species, available data suggest that SD studies and their applications for fish aquaculture should emphasize the complex nature of this trait and thus, using appropriate quantitative genetics tools for its study.

In fact, consistent variation among families has been reported on sex ratio in European sea bass (Vandeputte et al., 2007), turbot (Haffray et al., 2009; Martínez et al., 2009), Nile tilapia (Lozano et al., 2013) and zebrafish (Liew et al., 2012). In some species, the additive genetic component underlying SD or sex ratio was even estimated (Vandeputte et al., 2007; Lozano et al., 2013). In zebrafish, a polygenic SD system was suggested based on inter-family and inter-strain variation, and consequently several QTL at different genomic locations were identified, some of them associated to the different strains studied (Bradley et al., 2011; Anderson et al., 2012; Howe et al., 2013). In other species where major loci are involved, the application of QTL screening or genomic association analysis to look for the SD region demonstrated to be efficient and new SD-related genomic regions were identified (Martínez et al., 2009; Hermida et al., 2013), sometimes denoting important intraspecific variation such as in *Eigenmannia* or in cichlid species complex (Ser et al., 2010; Henning et al., 2011; Parnell and Streelman, 2013). As a consequence, selection has demonstrated to be efficient to change sex ratio in progenies of several species and in other related traits like the sensitivity to temperature on sex ratio (Baroiller et al., 2009; Liew et al., 2012; Lühmann et al., 2012; Lozano et al., 2013).

## SEX-ASSOCIATED TRAITS IN FINFISH AQUACULTURE: SOME RELEVANT EXAMPLES

In many fish species one sex grows faster or matures earlier than the other and these differences may be accentuated under aquaculture conditions (Breder and Rosen, 1966; Parker, 1992; Piferrer et al., 2012). Sex-associated growth differences generate size dispersion, and therefore, classification must be performed for feeding and to avoid cannibalism or size hierarchies affecting social relations (Dou et al., 2004). This represents more work in animal husbandry, and a higher number of production units to adjust different growth groups (Piferrer et al., 2012). Sexual growth dimorphism can favor males (e.g., tilapias) or, more frequently, females (flatfish, sea bass, among others). In some cases, as in the turbot, females can be 50% larger than males (Imsland et al., 1997). In other cases, as in the European sea bass, the rearing conditions result in highly male-biased stocks (Piferrer et al., 2005). A great deal of research towards the development of sex control methods has been carried out in fish (Piferrer, 2001; Cnaani and Levavi-Sivan, 2009).

Sex-associated markers are very useful in this context for precocious sex identification, especially in those species lacking morphological sexual dimorphism. This can aid to identify the sex of potential broods in genetic breeding programs and to avoid sex bias in the selected population. However, the most relevant application of sex-associated markers is to identify the genetic sex of sex-reversed individuals after hormonal treatment to accelerate the processes for establishing monosex populations (Penman and Piferrer, 2008). The availability of sex-associated markers or even better the SD master gene makes it possible to shorten this process using marker assisted selection (MAS) or gene assisted selection (GAS), respectively.

### TURBOT

The strong sexual growth dimorphism of turbot has promoted the interest of industry for all-female populations. No sex-associated karyotype heteromorphism have been detected in turbot, either after analyzing the mitotic or the 11-fold longer and higher resolution meiotic chromosomes (Bouza et al., 1994; Cuñado et al., 2002). This suggests that the SD region in turbot is small or not large enough to be detected with these cytogenetic techniques. A QTL screening performed with 100 homogeneously distributed microsatellites identified a major SD region in the proximal region of LG5 between two markers separated by 17.4 cM (Martínez et al., 2009). Assuming a single SD region with full penetrance, the SD master gene (SDg) was located at 2.6 cM of the Sma-USC30 microsatellite locus, representing 1.4 Mb according to the general relationship between genetic and physical maps in turbot (Bouza et al., 2007). Also, the analysis of segregation of Sma-USC30 in all families demonstrated that the mother is responsible for sex, supporting a ZZ/ZW system (Martínez et al., 2009) in accordance with the sex ratios observed in progenies from hormonal sex reversed parents (Haffray et al., 2009). However, sex association of Sma-USC30 showed variation among families (between 84 and 100%) and, in addition, other minor QTL were detected at LG6, LG8, and LG21. Temperature also showed some influence on sex ratios (Haffray et al., 2009), although without the general trend reported in most species where the proportion of males increased with temperature (Ospina-Álvarez and Piferrer, 2008).

**FIGURE 3:**
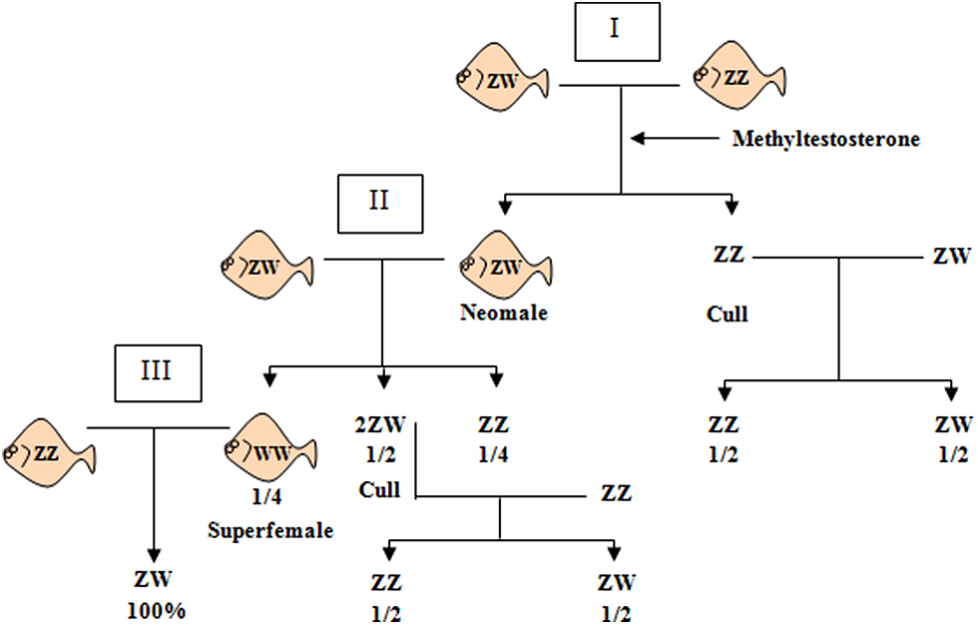
**Scheme of crosses aimed at obtaining all-female populations of turbot.** I, II, and III represent the three generations required for the full process. Neomales (ZW) are obtained in generation I by applying methyltestosterone in the diet in undifferentiated fry. Identification of neomales (I) and superfemales (II) is usually done by individual progeny testing, so parents producing 50:50 f/m in cross I and II are culled because they are not neomales and superfemales, respectively. Crossing normal males (ZZ) with superfemales (WW) would produce 100% females (ZW) assuming a single full penetrant SDg.

Using the Sma-USC30 marker, it was possible to classify correctly 98.4% of the individuals in four out of five families analyzed. This information was essential to develop a molecular tool for precocious sex identification in turbot, currently under a Spanish patent (Ref. number: 2 354 343**)**. Since sex cannot be identified in turbot until fish maturation, precocious sex identification is relevant in breeding programs to estimate sex ratio in selected progenies. This molecular tool is also essential to facilitate the achievement of all-female populations. Because turbot displays a ZW mechanism, getting all-female populations requires a three-generation pedigree starting from hormonal sex-reversed ZW neomales until obtaining WW superfemales in the progeny of the second cross (**Figure 3**). These superfemales would produce all-female offspring after being crossed with normal ZZ males. However, the chromosome constitution of ZW neomales or WW superfemales require individual progeny testing of hormone-treated larvae (ZZ or ZW) and of female offspring in cross II (ZW or WW).

Progeny testing is long and involves at least 2–3 years until fish are mature for checking sex ratio in their progenies. Additionally, sex can only be visually identified 4–6 months after hatching in sacrificed offspring, which globally represents a minimum of 2.5 years for progeny testing per generation. The availability of a genetic marker closely linked to the SDg has enabled assessment of the genotypic sex of fish just after 4–6 months when a fin clip can be obtained without damage, thus saving a minimum of 5 years for the production of all-female progenies. However, some limitations still remain, so this technology should be further refined. First, production of 100% females is not often fulfilled because turbot sex also depends on other minor genetic and environmental factors. In addition, because Sma-USC30 is a linked marker to the SDg, we need to establish association of SmaUSC-E30 to sex at family level because this is not a sex-specific marker. Finally, because crossovers can take place between the marker and the SDg, some of the selected ZW neomales or WW females would not show the expected genetic constitution. Nevertheless, this tool is being used by turbot companies with encouraging results.

The availability of a denser genetic map with around 600 markers (Bouza et al., 2012; Hermida et al., 2013), the enriched database with reproduction and immune genes (Pereiro et al., 2012; Ribas et al., 2013), and the recently assembled turbot genome (Figueras et al., in preparation) is enabling a more refined analysis of the SD region to identify the SDg, to analyze its relationship with the previous suggestive QTL, and to study the evolution of sex chromosomes (Taboada et al., 2014). The fine mapping performed has narrowed the genomic position of the SDg to a few kilobases and now much more genetic markers closely associated to the SDg are available, thus facilitating the precocious evaluation of sex. However, although several strong candidates were identified in that region (*sox2*,* dnajc19*,* and fxr1*), none of them were associated with sex at the species level, which illustrates the difficulty of such enterprise (Hermida et al., 2013). In addition, this work has provided additional support for the existence of minor factors at LG6, LG8, and LG21 in new families and demonstrated an interactive rather than an additive component between minor and the major SD QTL.

### EUROPEAN SEA BASS

The European sea bass is a gonochoristic marine teleost of the family Moronidae that is present in the NE Atlantic, from Norway to NW Africa and in the Mediterranean up to the Black Sea. As in most teleosts, sea bass does not show sex chromosomes (Devlin and Nagahama, 2002). Female homogamety (XX) has been ruled out since sex ratios of normal diploid and gynogenetic offspring are equivalent (Felip et al., 2002), and offspring from masculinized females is not female-biased (Blázquez et al., 1999). Other data from hormone-treated fish suggested that male homogamety with environmentally male-biased sex ratio would still be a possibility (Vandeputte et al., 2007). However, a polygenic sex determining system (Vandeputte et al., 2007) influenced by temperature (Piferrer et al., 2005) seems to fit better to data. Sex ratio shows interfamily differences with specific and measurable paternal and maternal components and at least two genes would be necessary to explain the variation pattern observed (Vandeputte et al., 2007), although no sex-associated QTL screening was carried out. The temperature can greatly influence European sea bass sex ratios (Piferrer et al., 2005) and a genetic component determining differential sensitivity to the masculinizing power of temperature has been reported (Saillant et al., 2002). Variation found on female percent due to thermal treatments show that genetic and environmental components are of comparable magnitude, supporting the notion of a continuum between GSD and ESD components of SD in the European sea bass.

In the European sea bass, gonads remain undifferentiated during post-larval stages until fish attain about 8 cm standard length, usually at about 5–6 months of age (Bruslé and Roblin, 1984; Blázquez et al., 1999; Navarro-Martín et al., 2009a; Díaz et al., 2013). European sea bass females differentiate earlier, are bigger, and mature later than males (Blázquez et al., 1999; Navarro-Martín et al., 2009a; Díaz et al., 2013). While still sexually undifferentiated, European sea bass gonads can be influenced by environmental abiotic factors or external factors such as sex steroids (Navarro-Martín et al., 2009a), but once sex is determined remains throughout life (Zanuy et al., 2001).

One of the main genes involved in fish GD as outlined above is the aromatase (cytochrome P450). This gene is present in two paralogous copies as a result of the teleost genome duplication, one predominantly expressed in the ovary (*cyp19a1a*; Dalla Valle et al., 2002) and the other in the brain (*cyp19a1b*; Blázquez and Piferrer, 2004). Interestingly, the *cyp19a1a* promoter exhibits important conserved binding sites for several genes of the GD network such as *sf1*, *sox*, *foxl2* or *ar*.

Recent work by Navarro-Martín et al. (2011) showed how temperature during early development is linked to the production of male-biased populations through differences in the methylation levels specifically on the gonadal aromatase promoter at one year. Different CpGs loci within the *cyp19a1a* promoter showed different sensitivities to temperature, suggesting a different role on regulation of aromatase expression. Methylation of gonadal aromatase promoter is thought to be the cause of the lower expression of aromatase in the temperature-masculinized fish linked to the suppressed ability of *sf1* and *foxl2* transcription factors to stimulate gonadal aromatase expression (Navarro-Martín et al., 2011).

While 15°C has been proposed as the optimal temperature for larval rearing in European sea bass (Koumoundouros et al., 2001), optimal growth in juveniles is found at 26°C and 13°C is considered as detrimental (Person-Le Ruyet et al., 2004). European sea bass hatcheries usually apply high temperatures (>20°C) to speed up growth rates, but male-bias progenies determine a loss of biomass. In the European sea bass the thermosensitive period includes from half-epiboly to mid-metamorphosis (∼17–18 mm total length; ∼70 dph; Koumoundouros et al., 2002), and treating fish with high temperatures masculinize a high proportion of the population (Navarro-Martín et al., 2009b), while temperatures never surpassing 17°C until metamorphosis yielded the maximum female proportion (Pavlidis et al., 2000). However, raising fish at low temperatures (15°C) for a long period also masculinize the population even more than a high temperature thermal treatment (Saillant et al., 2002). A thermal protocol to maximize the number of females was developed (Navarro-Martín et al., 2009a) and recently patented (patent no N200802927). Such protocol consisted on maintaining 17°C water temperature until the end of the thermosensitive period, and then increasing the temperature as a ratio of 0.5°C/day until 21°C to allow high growth rates. It should be stated that there is no temperature regime that will increase the proportion of females in the European sea bass. Instead, proper management of temperature during early development will avoid induced masculinization by applying high water temperatures before the thermosensitive period is over. What a proper thermal regime does is allowing the production of as many females as the polygenic system will allow, bearing in mind the sex ratio of a given brood, even reared at the optimal thermal regime, will ultimately depend on the genetic constitution of both parents.

With this information at hand, different strategies have been devised by European sea bass industry to improve growth rate. On one hand, classical breeding programs may incorporate sex ratio as additional phenotypic information to get female-biased progenies. This strategy would be much more efficient if sex-related QTL screening were performed and sex-associated genetic markers explaining an important proportion of trait variance were incorporated following MAS selection programs. On the other hand, controlling sex-ratio through larval rearing temperature protocols could increase female proportions by avoiding masculinization due to elevated water temperature. Currently, efforts are underway aimed at selecting broodstock that will produce the highest number of females and investigating the possibility that, among these fish, those that are more resistant to the masculinizing temperature can also be selected. An additional step would be further investigation of the epigenetic mechanisms responsible for the inheritance of the high-temperature masculinization, as already done in the half-smoth tongue sole (Chen et al., 2014). In the case of the European sea bass, the goal would be the opposite, i.e., to select as future broodstock those fish that despite being reared at high (masculinizing temperature) they do not become masculinized. In this way, the production of the highest number of females across different generations perhaps could be achieved.

### SALMONIDS

The Salmonidae family (11 genera, 70 species; salmon, trout, char, whitefishes, and graylings) includes several of the most economically important species for aquaculture and fisheries industry (the third largest world fish production). Salmonids are worldwide distributed and some species have played a vital role in the life and culture of the North hemisphere societies for thousands of years.

Atlantic salmon (*Salmo salar*) is the leading intensively farmed marine fish. In 1998 global farmed production exceeded the world’s total wild salmon captures, and in 2010 around 1.2 million tons were produced worldwide (Food and Agriculture Organization [FAO], 2014). Atlantic salmon breeding companies have achieved more than 100% growth increase in around six generations of selection, and significant improvement in disease resistance and delay at the onset of sexual maturation. The vast majority of farmed Atlantic salmon eggs and smolts are now sourced by such breeding companies (Bostock et al., 2010). Another important salmonid species, the rainbow-trout (*O. mykiss*), is the most-widely cultivated cold fresh water fish species in the world.

In salmonids, early maturation occurs differentially in males and females and is responsible for some problems related to intensive culture (Felip et al., 2006) including reducing growth, increasing disease susceptibility and changing of organoleptic properties. Thus, in this group, females are preferred for production. In all salmonid species investigated so far, SD is strictly genetic with a male heterogametic sex-determination system (Davidson et al., 2009), although no heteromorphisms or only slight morphological differentiation have been reported associated to sex chromosomes in some species. In rainbow trout, all-female production is generalized in Europe since females are still immature at harvest. Neomales (hormone sex-reversed genotypic females) are used to produce all-female progenies from XX neomale crossed to XX females. Triploids are also produced using chromosome set manipulation techniques to avoid sexual maturation for the production of individuals of bigger size (more than 3 kg; Piferrer et al., 2009).

Both, endocrine and genetic technologies have been implemented for sex control on a production scale in salmonids. Nevertheless, the phenotypic differentiation of males and females is still problematic until the fish become sexually mature and, thus, in some species it is still necessary a genetic/molecular test for sexing. Sex-specific markers, including a linked sequence to the growth hormone pseudogene, have been developed in the last decade for Pacific species of the *Oncorhynchus* genus, rainbow trout (*O. mykiss*), Chinook salmon (*O*. *tshawystscha*), coho salmon (*O. kisutch*), chum salmon (*O. keta*), and pink salmon (*O. gorbuscha*; Brunelli and Thorgaard, 2004).

Recently, the *sdY* (sexually dimorphic on the Y chromosome) was identified as a male-specific linked gene on the Y chromosome in most salmonids (Salmoninae, Coregoninae and Thymallinae subfamilies), strongly suggesting that *sdY* may be the conserved master sex-determining gene of this group (Yano et al., 2012, 2013). However, because this gene is not located at homologous genomic positions among the different salmonid species, it has been suggested its jumping associated to mobile elements (Yano et al., 2013). Irrespective of its location, sequences of this gene may represent a useful tool for sexing. However, some exceptions were observed to this general rule, and in the Coregoninae subfamily, while *Stenodus leucichthys* showed *sdY* as a male specific gene, *Coregonus lavaretus* and *Coregonus clupeaformis*, both males and females contain the *sdY* gene, so a different SD mechanism appears to be involved.

The analysis of sex-associated markers in several families of the SALTAS Tasmanian Atlantic salmon program from different male lineages allowed the discovery of three sex-associated markers (Ssa03, Ssa06, and Ssa2) mapping at three different linkage groups. Ssa2 is the same sex-associated marker previously reported in Atlantic European populations (Eisbrenner et al., 2014), but Ssa03 and Ssa06 represent new genomic positions. These three loci showed positive amplification for *sdY* gene and most individuals analyzed showed a good concordance between phenotypic sex and *sdY* PCR amplification, suggesting the movement of *sdY* to new positions within this species. However, some inconsistencies were detected among the sex marker associated genotypes, the presence of *sdY* gene and the phenotypic sex. Based on these findings, and in the fact that the sdY protein lacks a DNA binding domain, these authors suggested the existence of another sex determining gene in Atlantic salmon upstream to *sdY*. Also, they emphasized the importance of using many families to identify sex-associated markers in salmonid species (Eisbrenner et al., 2014). Furthermore, the recent characterization of the male-specific region on the Y chromosome of rainbow trout which contains the *sdY* gene and the male specific marker (OmY1), revealed several male specific SNPs associated with 12 single-copy protein coding sequences whose role in the SD should be further analyzed (Phillips, 2013). Data show that even in a species with an apparent well-established SD mechanism variation is observed. Thus, caution should be taken when applying sex-associated markers for establishing associations and also to for their application to precocious sex determination.

### TILAPIA

The tribe Tilapiini includes more than 80 species of cichlid fish (family Cichlidae, order Perciformes). Tilapias are endemic to Africa and the Middle East, but they have been introduced into most tropical and subtropical countries for aquatic weed control and aquaculture. Tilapia culture was considered a resource to improve protein supply in developing countries, but nowadays there is also an important market for tilapias in Japan, United States, European Union as well as in other developed countries. Tilapia is one of the fastest growing fish farming sector, being China the production leader, and it constitutes the second most important group of farmed fish after carps and the most widely grown of any farmed fish (85 producer countries). The main aquaculture species is the Nile tilapia (*O. niloticus*) with a production exceeding 3.2 million metric tons in 2012 (Food and Agriculture Organization [FAO], 2014).

Tilapias exhibit an important sexual growth dimorphism in favor of males. Additionally, tilapias show early maturation (e. g., 4–5 months old in Nile tilapia), which determines successive spawning during the growing period, leading to stunting of growth (Beardmore et al., 2001). All these circumstances make it difficult to establish a uniform product, and all male fry are preferred.

Monosex culture can be obtained by different approaches: manual separation of males and females; hybridization between species to produce all-male offspring; or artificial sex reversal using hormones. The most frequent method for producing all-male populations in tilapia was the treatment with 17α-methyltestosterone included in the diet of sexually undifferentiated fry. If properly applied, farms can produce male populations with 98 to 100% effectiveness. However, marketing of hormonally treated fish can also be a problem for health and the direct use of hormones is usually forbidden by food safety regulations in European Community, although in other countries it may be allowed. One way to get through this problem is to combine a method for sex reversal with a breeding scheme aimed at obtaining broodstock that produces monosex fry following the reverse procedure as outlined before for turbot (**Figure 3**). In the case of Nile tilapia, it is necessary to produce YY supermales by crossing neofemales (XY) with regular males (XY). As in turbot, the use of sex-linked DNA markers could shorten the process by distinguishing XX, XY, or YY individuals, thus avoiding the identification of individual genotypes by progeny testing.

Sex chromosomes are not identifiable in tilapias using standard cytogenetic techniques. Most species show 22 chromosome pairs but there is no a heteromorphic sexual chromosome pair. Association studies suggested that sex is determined in tilapias by the existence of major genes located at linkage groups 1, 3, and 23 in the different species (Cnaani et al., 2008; Cnaani, 2013). Additionally, the heterogametic sex can be either the male or the female, depending upon the species, and ZZ/ZW (LG3) and XX/XY (LG1) systems have been reported within the same genus, i.e., in *O. niloticus and Tilapia zillii* (XX/XY), and in *Tilapia mariae, Oreochromis aureus, Oreochromis karongae, Oreochromis tanganicae* (ZZ/ZW). Some families of blue tilapia have been found segregating for both loci, and in these cases the ZW locus appears to be epistatic over the XY (ZW/XY individuals are female; Lee et al., 2004). In Mozambique tilapia (*Oreochromis mossambicus*), the SD locus was found at LG1 (Liu et al., 2013). However, Cnaani et al. (2008) found sex-associated markers on LG1 and LG3 in three families of this species. These discrepancies may be determined by the different genetic background of families and strains used in these studies (Liu et al., 2013). The differences in SD within species show that minor genetic factors segregate and interact with major genes in addition to the influence of environment factors (Baroiller et al., 2009), suggesting that SD should be treated as a quantitative trait and its dissection approached using QTL screening (Eshel et al., 2012).

The main cultured species is the Nile tilapia (GIFT project; Lozano et al., 2013). Most data pointed to LG1 as the sex chromosome in this species (Cnaani et al., 2008) and recently a small candidate region was narrowed by RAD (restriction site associated DNA) sequencing on a 1–2 Mb region on LG1 (Palaiokostas et al., 2013a). However, other sex-linked markers have been identified in *O. niloticus* and its hybrids (*O. niloticus x O. aureus*) mapping on LG3 and LG23 (Eshel et al., 2012). Also, it has been shown by linkage analysis that genetic factors are involved in the sensitivity of SD to temperature and that these factors are located depending on families on the same chromosomal regions as the major QTL at LG1, LG3, and LG23 (Lühmann et al., 2012).

The closely sex-associated genomic region identified on LG1 includes 10 genes not previously related to SD in other species, and two SNPs probed to be very useful for sexing individuals, thus being worthy for production all male stocks by industry (Palaiokostas et al., 2013a). However, some of these markers were not associated to sex in different strains or families, so checking several markers on different linkage groups should be done before its application. Most studies on SD in *O. niloticus* have been carried out on fish derived from Lake Manzala in Egypt population, but today this species show a worldwide production and new information is arising which probably will confirm the necessity for checking a set of markers previous to sexing specific strains.

## CONCLUSION

Major genetic factors can explain a high proportion of the SD variance in fish in accordance with the hierarchical gonad development of vertebrates and with the models proposed to explain its origin and evolution. However, several other minor genetic and environmental factors also influence sex following a complex interactive pattern. Thus, the currently available information supports the idea that sex can be regarded as a complex trait in fish, with the influence of one or more genetic factors in addition to possible environmental influences, depending upon the species. The presence of genetic factors regardless of whether SD is under the control of a master gene, a polygenic system or driven by an environmental factor enables their application in MAS programs to exploit the benefits of a particular sex.

## Conflict of Interest Statement

The authors declare that the research was conducted in the absence of any commercial or financial relationships that could be construed as a potential conflict of interest.
